# Response to Comments on Zheng et al. “Association between Promoter Methylation of Gene *ERCC3* and Benzene Hematotoxicity” *Int. J. Environ. Res. Public Health* 2017, *14*, 1393

**DOI:** 10.3390/ijerph14121529

**Published:** 2017-12-07

**Authors:** Caihong Xing, Min Zheng

**Affiliations:** Key Laboratory of Chemical Safety and Health, National Institute for Occupational Health and Poison Control, Chinese Center for Disease Control and Prevention, Beijing 100050, China; zhengm0916@126.com

We would like to thank Moshammer and Poteser for their comments. We agree with Moshammer and Poteser’s suggestion that epigenetic changes do lead to genetic instability and the methylation of promoter region of *ERCC3* is likely involved in the genotoxic effects pathway of benzene. Epigenetic effects of benzene need more complex explanation in causative pathway leading to hematoxicity.

Regarding whether the methylation pattern is specific for benzene, Bollati et al. [1] found the hypomethylation of melanoma antigen-1 (*MAGE-1*) cancer associated antigen gene and hypermethylation in *p15* in workers exposed to benzene. We reported downregulation of *p15* and *p16* expressions were associated with DNA promoter hypermethylation in benzene poisoning (BP) patients [2]. Induction of hypomethylation had been demonstrated to be induced by benzene metabolite hydroquinone in vitro in TK6 cells [3]. These findings show that benzene exposure may induce aberrant methylation patterns which were found in malignant cells and suggested that these epigenetic alterations may be another possible mechanism of transcriptional dysregulation underlying the leukemogenicity of benzene. The functional relevance of the two CpG units in *ERCC3* promoter region should be explored in a well controlled in vitro system to better understand the effects of benzene-induced hypermethylation on gene dysregulation.

In this study, we only observed an association between lower neutrophil and higher methylation in exposed workers. In a study of 250 benzene-exposed workers and 140 unexposed controls, single-nucleotide polymorphisms in genes (*BLM*, *TP53*, *RAD51*, *WDR79*, and *WRN*) that play a critical role in DNA repair and genomic maintenance were associated with highly significant reductions in the white blood cell count among benzene-exposed workers but not controls [4]. Another study had the same findings that the length of service was negatively associated with WBC counts among benzene-exposed workers but not controls. In the present study, we found that there was no correlation between the levels of CpG methylation and neutrophil counts when the analysis was restricted to controls or applied to the whole group. Therefore, we propose that this correlation could be a result of benzene exposure. Aberrant methylation has been shown to be an early event in carcinogenesis [5,6,7] and the reduction of neutrophil count is one of the early clinical symptoms after benzene exposure. Therefore, our findings suggest that the negative correlation between the methylation level and neutrophil count may contribute to the functional changes at the early stage of benzene exposure. Future studies are warranted to determine how these early changes on neutrophil, possibly hematopoietic stem and progenitor cells, lead to the development of leukemia.

We agree that it is necessary to study hypermethylation patterns in a longitudinal manner starting after an episode of toxic neutropenia. We analyzed the methylation levels in the BP group compared with the non-BP group and found that there was no statistical significance between these two sub-groups. The toxicity of benzene discussed in this paper did not specifically apply to the BP group but rather to all benzene-exposed workers including BP. Although the BP workers had their poisoning episode approximately 20 years ago and afterwards were no longer exposed, they still display lower nuetrophil counts compared to the other exposed workers (2.55 ± 0.84 × 10^9^/L vs. 3.43 ± 1.04 × 10^9^/L, *p* < 0.01). It is necessary to verify the correlation between methylation of *ERCC3* gene and hematotoxicity in a larger cohort of benzene-exposed population.

We are thankful to Moshammer and Poteser for their kind suggestion. We thank the editor for giving us the opportunity to provide a reply to the letter.
